# Adjunctive effect of collagen membrane coverage to L-PRF in the treatment of periodontal intrabony defects: a randomized controlled clinical trial with biochemical assessment

**DOI:** 10.1186/s12903-023-03332-0

**Published:** 2023-09-04

**Authors:** Ramy Mubarak, Doaa Adel-Khattab, Khaled A. Abdel-Ghaffar, Ahmed Youssef Gamal

**Affiliations:** 1 Department of Oral Medicine, Periodontology and Diagnosis, Faculty of Oral and Dental Medicine, Future University, Cairo, Egypt; 2 Department of Oral Medicine, Periodontology and Diagnosis, Faculty of Dentistry, Ain Shams University, Cairo, Egypt; 3 Oral Medicine, Periodontology and Diagnosis, Faculty of Dentistry Ain Shams University, Cairo, Egypt; 4 Faculty of Dentistry, Misr University for Science and Technology, Giza, Egypt

**Keywords:** Periodontitis, Periodontal guided tissue regeneration, Platelet-rich fibrin, Platelet-derived growth factor, Vascular endothelial growth factors, Centrifugation

## Abstract

**Background:**

The innovation of leukocyte platelet-rich fibrin (L-PRF) has added enormous impact on wound healing dynamics especially the field of periodontal regeneration. The release of growth factors (GF) is thought to improve the clinical outcomes in infrabony defects. The aim of this study was to evaluate the clinical effect of covering L-PRF contained infrabony defects with collagen membranes (CM), and to compare their GF release profile to uncovered L-PRF defects and open flap debridement (OFD).

**Methods:**

Thirty non- smoking patients with infrabony pockets participated to be randomly assigned to OFD group (*n* = 10), L-PRF group (*n* = 10), or L-PRF protected CM group (*n* = 10). Plaque index (PI), gingival index (GI), probing depth (PD), clinical attachment level (CAL) and the radiographic defect base fill (DBF) were measured at baseline and at 6 month following surgical intervention. Gingival crevicular fluid samples were obtained on days 1, 3, 5, 7, 14, 21 and 30 days following surgery for the Platelet Derived Growth Factor-BB (PDGF-BB) and Vascular Endothelial Growth Factors (VEGF) release profile evaluation.

**Results:**

For all patients, a statistically significant (P ≤ 0.05) reduction in PI, GI, PD and CAL were reported throughout the study period. Differences between the three treatment modalities were not statistically significant. PRF + CM showed a statistically significant DBF compared to OFD and L-PRF groups at follow up. Quantitative analysis of PDGF-BB and VEGF levels demonstrated a statistically significant (*P* < 0.001) decline between measurement intervals for all groups with no statistically significant differences between the three groups.

**Conclusion:**

Within the limitations of this study, L-PRF coverage with CM may augment defect base fill through its mechanical protective effect without enhancement in the release profile of VEGF and PDGF. The non-significant intergroup differences question the validity of the claimed extra physiologic concentration of GF offered by L-PRF harvests.

**Trial registration:**

The present study was registered at ClinicalTrials.gov (NCT05496608), (11/08/2022).

**Supplementary Information:**

The online version contains supplementary material available at 10.1186/s12903-023-03332-0.

## Introduction

Periodontitis is a chronic inflammatory wide- spread infection with exceptionally offensive mutilation of the periodontium. Regenerative periodontal surgery aims to restore periodontal tissues lost during progression of the disease. For vertical bone defects regenerative interventions included the use of enamel matrix derivatives, Nd:YAG laser, bone grafts, guided tissue regeneration (GTR), and biologic modifiers to achieve regeneration [1]. Guided tissue regeneration (GTR) utilizes a barrier membrane for bio- exclusion of unfavorable cells and allowing for restoring the harmed periodontal tissues that were lost [2]. Biologic modifiers on the other hand, target tissue engineering strategies and protocols including platelet concentrates and growth factors (GF) to amplify the regenerative potential within the periodontal lesion [3].

Naturally, activated platelets in the wound clot secrete coagulation factors, cytokines, and GF to orchestrate the physiological events of wound healing [4]. Of particular interest, Platelet-derived growth factors (PDGFs) regulate the migration, proliferation, and survival of the mesenchymal cells and enhance extra cellular matrix remodeling via promotion of collagen production [5]. In addition, Vascular endothelial growth factors (VEGF) stimulate angiogenesis within tissues, new vessel formation, and eventually increase tissue perfusion and nutrient supply [6]. In contrast to the short half-life of the naturally released GF, platelet concentrates served as successful biologic modifiers providing higher concentrations for longer duration. Among various generations of platelet concentrates, Platelets rich fibrin (PRF) possesses a three-dimensional fibrin scaffold trapping numerous autologous platelets, neutrophils, and macrophages; the result is a biomaterial with an antibacterial features and considerable reservoir of a sustained and gradual release of GF over time [7].

Both GTR and PRF have their limitations in the literature; Despite collagen membranes (CMs) used in GTR do not require removal from the wound site and could retain their structural identity throughout the degradation process, several obstacles exist that pose GTR technique to collapse in clinical situations including contamination, uncontrolled degradation rates, uncontrolled barrier function, and mechanical collapse of the membrane. Most importantly, it possesses a limited capability for regeneration [8]. Consequently, most regenerative studies concentrate on either improving membrane features, [9] or enhancing the wound healing dynamics (e.g., platelet concentrates). Regarding L-PRF, biochemical quantification of GF release was documented in the literature however, most studies were in vitro and ex- vivo models that neglect the open wound nature and the complex microbial challenge in periodontal lesions. Consequently, some clinical trials demonstrated that L-PRF and L-PRF blocks can sustain the release of GF for 7–14 days [10], contrary to others who reported no significant differences in GF levels between L-PRF enhanced periodontal wounds and non L-PRF biomaterials [11].

In the present study, it was speculated that CM can be used to preserve the GF concentrations released by L-PRF enhancing the clinical and radiographic outcomes in treating infrabony pockets. Therefore, the aim of the present study was to investigate the clinical and radiographic outcomes for infrabony defects treated with L-PRF with and without CM coverage and to evaluate the secretory profile of PDGF and VEGF during early stages of periodontal tissue healing.

## Materials and methods

The study protocol was following the Declaration of Helsinki (revised in October 2018) and was approved by the Research Ethics Committee of the faculty of Dentistry, Ain Shams University (FDASU-RecD061808). A total number of thirty patients undergoing periodontal therapy were enrolled in this prospective cohort randomized clinical and biochemical trial consecutively from the outpatient clinic of Periodontology Department, Faculty of Dentistry, Ain Shams University, Cairo-Egypt between January 2020 to November 2021. Participants were informed verbally and written informed consent was obtained.

### Eligibility criteria

Patients enrolled in this study were diagnosed with stage III, grade B periodontitis. Inclusion criteria included the presence of a minimum of one interproximal pocket probing depth (PD) of ≥ 6mm and ≥ 5 mm clinical attachment level (CAL) after 4 weeks from phase I therapy, and the presence of 2 or 3 osseous wall interproximal intrabony defects that are ≥ 3mm in depth. The diagnostic defect depth was measured as the distance from the interproximal alveolar crest (AC) to the base of the defect (BD) using an intraoral periapical radiograph. Exclusion criteria were systemic diseases or conditions that contraindicate periodontal surgeries and/or affect the formed elements of the blood, patients who received antibiotic therapy and/or anti-inflammatory drug within the past 6 months, vulnerable groups such as mentally challenged individuals, the presence of deleterious habits, interdental craters and 1 wall osseous defects. Pregnant females, smokers and patients with inadequate oral hygiene (Plaque index > 1 after phase one therapy) at the time of reevaluation of phase I therapy were excluded.

### Presurgical phase and treatment allocation

**Fig. 1:**
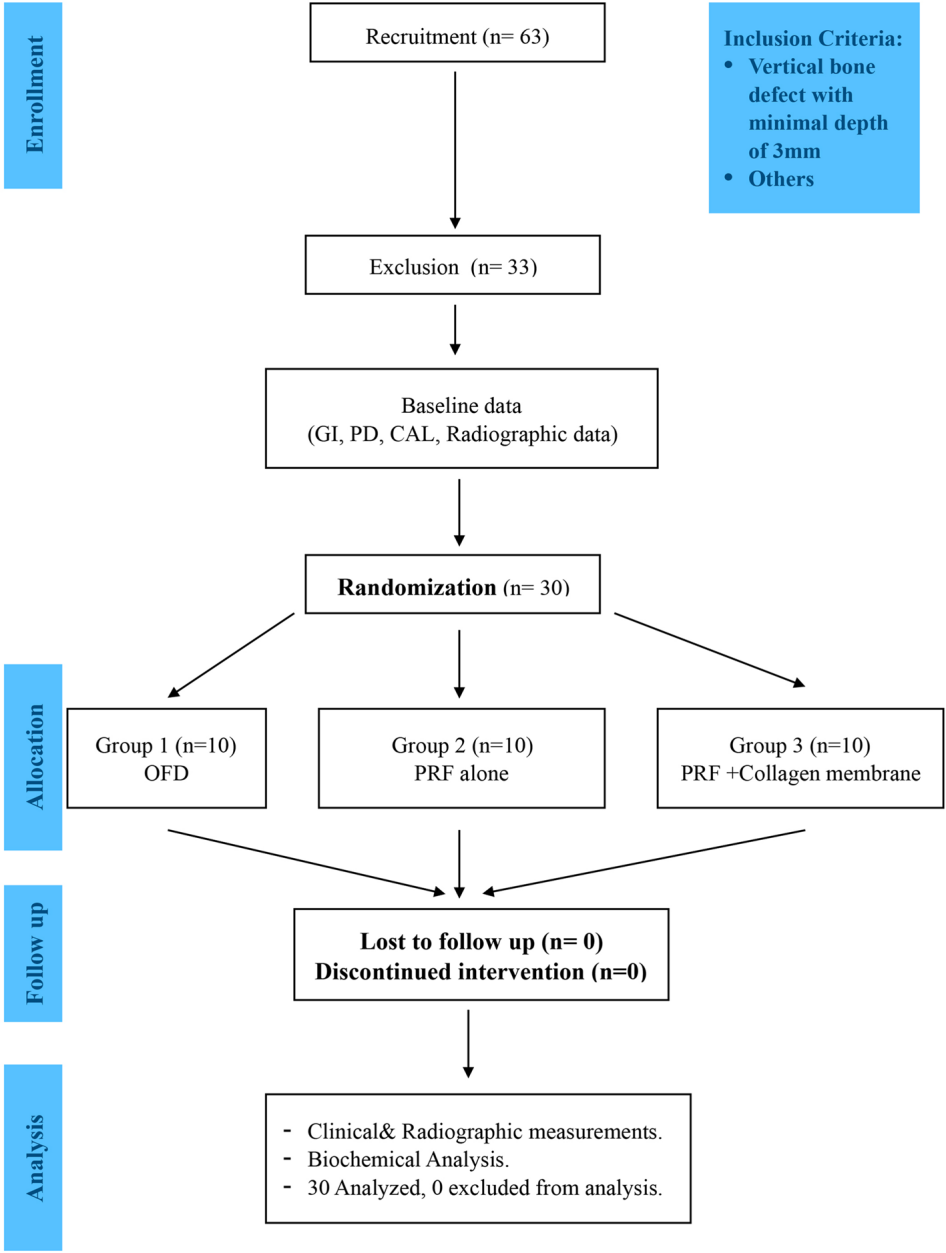
Consort checklist diagram

The three treatment groups were randomly allocated by a predetermined computer-generated randomization list.^1^ Allocation concealment was ensured using a sealed coded envelope containing treatment of the subject. Each patient was randomly assigned to OFD (control group): included intrabony defects treated by OFD. L-PRF: open flap debridement with L-PRF application in the infrabony periodontal defect was performed. L-PRF + CM: intrabony defect coverage with L-PRF followed by CM over coverage (Fig. 1). The outcome assessor (D. A-K) and the statistician were blinded in contrast to the skilled surgeon (R. M.) and patients as the performed interventions were completely different.

Criteria to confirm eligibility to surgical intervention included: 1) Persistent interproximal PD > 6 mm (distance from base of pocket to gingival margin); 2) CAL > 5 mm (distance from base of pocket to cemento-enamel junction (CEJ); and 3) interproximal DBF > 3 mm. The baseline data for the following clinical and radiographic parameters were recorded with the aid of an acrylic stent: (i) plaque index (PI); (ii) gingival index (GI); (iii) PD; and (iv) CAL. Clinical measurements were recorded using a graduated UNC-15 periodontal probe.^2^

### Surgical procedures

**Fig. 2:**
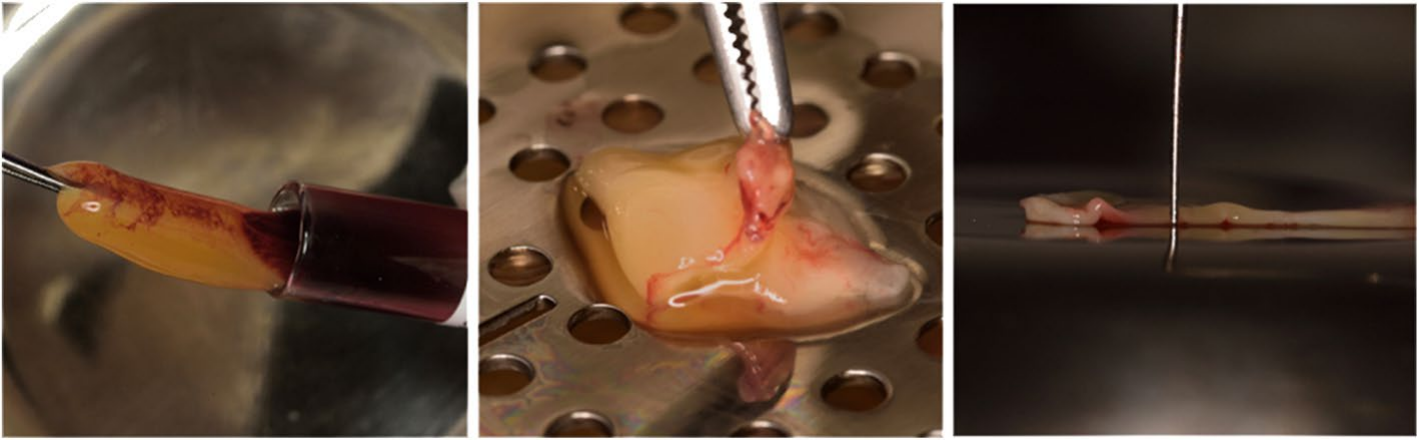
Preparation of L-PRF (**a**) L-PRF harvest removed from 10 ml plastic tube after centrifugation (**b**) The fibrin clot being removed from the tube are folded for compression. (**c**) Following compression of L-PRF membrane

All interventions were performed after administration of an adequate local anesthesia^3^ (2% lignocaine hydrochloric acid with epinephrine (1:100,000) using infiltration or nerve block techniques. For the test groups, L-PRF preparation was performed as follows (Fig. 2); blood sample was drawn via a standard venipuncture into 10 mL tubes^4^ without the addition of anticoagulants. Immediate centrifugation of the tubes was carried out at 2700rpm (408g) for 12 minutes utilizing a table centrifuge.^5^ The L-PRF harvest was held with a tissue forceps in the silica tube and the red element was cut off us-ing Tc Goldman fox scissors supplied with the PRF Kit^6^. The harvest was then transferred to the un-der-board surface of the L-PRF metal box. Each L-PRF harvest was folded over itself and compressed onto itself into 4 multiple layers. After applying each fold using the tissue forceps, the supplied press board of the box was placed on the membrane for compression. The latter applies gentle compression on the L-PRF clot through gravity without any active loading application by the operator.

For all groups an envelope flap was performed through sulcular incisions extending two teeth mesially and one tooth distally followed by mucoperiosteal flap reflection. Debridement of the periodontal lesion was then performed as well as planing of the exposed root surfaces. Following copious saline irrigation, reassessment of the bone defect morphology was performed and recorded. Being in a soft membranous form, the folded L-PRF harvest was easily grasped with tissue forceps and placed within the intrabony defect. The supplied PRF compactor was then used to tuck the L-PRF membrane within the defect up to the level of interproximal alveolar crests for L-PRF and L-PRF+CM groups. For L-PRF+CM, CM^7^ were trimmed to confine with the size of the bony lesion. The barrier membrane was placed and adapted over the defect area in such a form that the entire defect and ≥2 to 3 mm of the surrounding alveolar bone were entirely covered to prevent membrane collapse within the defect.

**Fig. 3:**
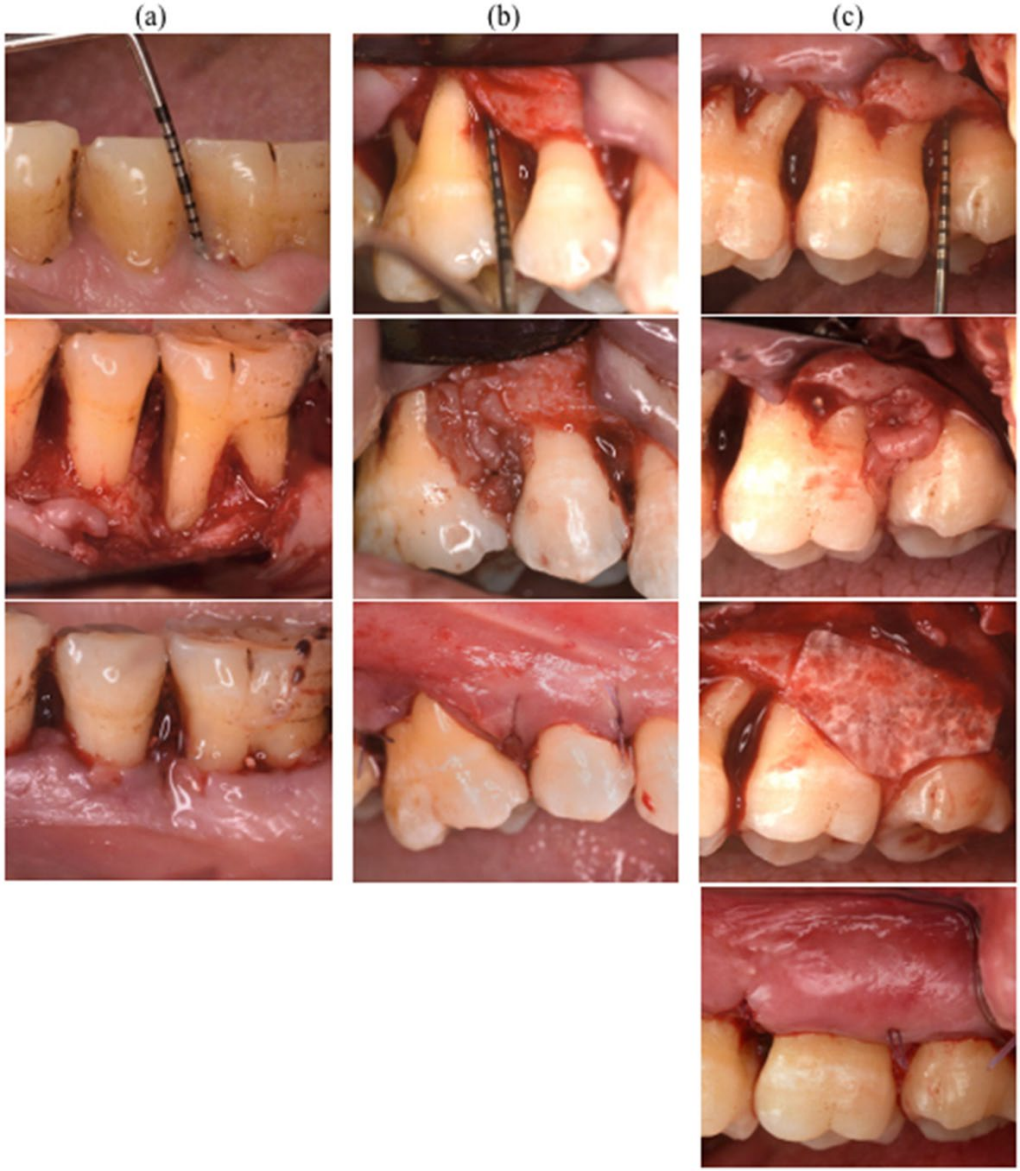
Presentation of a study patient for every group (**a**) Control site showing open flap debridement of the defect (left above) and wound closure (left below). (**B**) Second group (middle from above to below), L-PRF applied in the infrabony defects. (**C**) Third group (Right above to below) L-PRF was applied in the defect and protected with collagen membrane

Primary closure of the flap was performed with 5–0 Polypropylene sutures using internal vertical mattress design. A demonstration for the surgical protocol for every group is shown in Fig. 3. Post operative medications of analgesics (Ibuprofen 400 mg thrice daily^8^), and Antimicrobials (Amoxicillin trihydrate^9^ 500 mg thrice daily for 7 days) were prescribed for all patients. Patients were asked to cease mechanical oral hygiene measures at the surgical site for one week, and to rinse with 10 mL of 0.2% chlorhexidine gluconate mouth-rinse^10^ for two weeks. They were also instructed to report any unfavorable incidents such as pain, swelling, and bleeding from the surgical site. Patients returned at days 3, 5, 7, 14, 21, and 30 days for obtaining crevicular fluid samples as well as at the 6th month following surgery for clinical and radiographic follow up measurements.

### Outcome measure

A single masked examiner performed all of the following clinical measurements at baseline and 6 months after surgery: (i) Gingival index (GI); (ii) Plaque index (PI); (iii)Pocket probing depth (PD); and (iv) Clinical attachment level (CAL). For obtaining the radiographic images, a size 2 digital sensor^11^ was activated for 30 to 120 s, then the radiographic image was captured to appear on the computer monitor using operating parameters at 60 kVp, 7 mA and 0.2^12^ second exposure time via the long cone paralleling technique and using specialized holders. After images were captured, calibration and analysis were performed using DabiAtlante^13^ software. Two measurements were obtained as follows: (i) Defect Base Fill (DBF) being the highest coronal point at the base of the bony defect in which a continuous width of the periodontal ligament space was detected; (ii) Cemento enamel junction to the alveolar crest (CEJ- AC): where the AC represents the highest detected point on the interproximal bone.

Patients were asked to fast the day following surgery between 8:00 pm and 10:00 am to avoid irritation. On the day following surgery, three Gingival Crevicular Fluid samples were collected using filter paper (Periopaper^14^) from the same site by a single blinded examiner. Isolation and dryness of the field was first ensured using cotton rolls. The filter paper was grasped from the orange terminal and applied within the gingival sulcus at the mesiofacial line angle to a depth of 2 mm calculated from the gingival margin and left in place for 60 s. After removal, absence of blood or plaque on the paper surface was ensured. It was immediately transferred into safe- lock Eppendorf tubes diluted in saline solution (50 L). Samples were tagged with labels, preserved in a dark container and stored at 76C. Samples were collected on days 1, 3, 5, 7, 14, 21 and 30 days following surgery for PDGF-BB and VEGF level measurements using enzyme-linked immunosorbent assay kit.^15^

### Statistical analysis

Calculation of sample sizes using Epicalc program version 1.02 suggested that a minimum of 10 experimental sites were required for each of the three groups (The power was kept at 0.95, a significance level α = 0.05 (type I error), and an effect size of 0.825).

All measurements were entered and tabulated in Microsoft Excel, 16 and statistical analysis was performed with SPSS 20, 17 Graph Pad prism^18^ and Microsoft Excel 2016. The primary efficacy parameters were the clinical and radiographic measurements at baseline and 6 months following surgical intervention. Whereas the secondary efficacy parameters were PDGF-BB and VEGF levels. A post-hoc power analysis was made to obtain power of the study. A power analysis was conducted to ensure sufficient statistical power to test the null hypothesis of no difference among the various groups regarding the clinical and radiographic measurements (primary outcomes). The mean values of 6.1, 6.8, and 6.1 (probing depth (PD) and standard deviation (SD) of 0.4, were obtained from a previous study.^11^ These values were used to compute the effect size (f), which was determined to be 0.825 By employing the latter with an alpha (α) level of 0.05, a desired power of 0.95, and a number of groups of 3. Numerical data were presented as mean ± SD values and the data were evaluated for normality utilizing Kolmogorov–Smirnov and Shipro-Wilk tests. PD, CAL, PI, and GI data showed non-normal distribution, Kruskal Wallis test used to compare between tested groups. Wilcoxon singed rank test used to compare between follow-up periods. For PDGF and VEGF, data showed normal distribution, so repeated measures ANOVA used to compare between tested groups and follow-up periods followed by multiple comparisons with Bonferroni adjustment. The significance level was set at *P* ≤ 0.05.

## Results

The current investigation is a prospective clinical trial which involved 30 subjects (12 males and 18 females, mean age 38.75 years) assigned to: OFD (*n* = 10); L-PRF (*n* = 10); L-PRF + CM (*n* = 10). Baseline data, intervention and follow up were carried out between January 2020 and November 2021. The demographic characteristics of the study participants are shown in (S Table 1). During the study, no dropouts were reported, and all surgical sites demonstrated uneventful healing without any wound site infection or flap dehiscence. All subjects completed the follow-up visits and complied with the study recall appointments. Table 1 shows the statistical summary of the clinical and radiographic outcomes using mean and standard deviation of GI, PI, PPD, CAL, CEJ-BD, and CEJ-AC.

**Table 1:** Mean, standard deviation and comparison of groups for clinical and radiographic outcomes

Parameter	Time period	OFD (*n* = 10)	L-PRF (*n* = 10)	L-PRF + CM (*n* = 10)	Intergroup difference (*P-*value)
PI	Baseline	0 ± 0	0 ± 0	0 ± 0	1.00 NS
	6 months	1.3 ± 0.67	1.1 ± 0.74	1.1 ± 0.74	0.77 NS
	***P-*value (Intragroup)**	0.006*	0.009*	0.009*	
GI	Baseline	0 ± 0	0 ± 0	0 ± 0	1.00 NS
	6 months	1.3 ± 0.67	1.2 ± 0.79	1.1 ± 0.74	0.830 NS
	***P-*value (Intragroup)**	0.006*	0.010*	0.009*	
PD	Baseline	7.5 ± 0.97	7.5 ± 1.08	7.1 ± 1.1	0.617 NS
	6 months	3.7 ± 0.67	3.7 ± 0.82	3.2 ± 1.14	0.325 NS
	***P-*value (Intragroup)**	0.004*	0.004*	0.005*	
	**Percentage change**	-50.6	-50.6	-54.9	
CAL	Baseline	5.5 ± 0.97	5.3 ± 0.95	5 ± 1.15	0.616 NS
	6 months	2.4 ± 0.7	2.3 ± 0.67	2.4 ± 1.51	0.910 NS
	***P-*value (Intragroup)**	0.004*	0.005*	0.0015*	
	**Percentage change**	-56.3	-56.6	-53	
Radiographic CEJ-BD	Baseline	5.93^a^ ± 0.59	6.34^a^ ± 0.57	6.50^a^ ± 0.79	0.15 NS
	6 months	5.50^a^ ± 0.48	5.54^a^ ± 0.67	4.38^b^ ± 0.89	0.001*
	***P-*value (Intragroup)**	0.001*	0.0001*	0.001*	
	**Percentage change**	-7.25	-12.6	-32.6	
Radiographic CEJ-AC	Baseline	2.35 ^a^ ± 0.50	2.65 ^a^ ± 0.52	2.55 ^a^ ± 0.51	0.41 NS
	6 months	2.50 ^a^ ± 0.51	2.89 ^a^ ± 0.52	2.74 ^a^ ± 0.50	0.24 NS
	***P-*value (Intragroup)**	0.009 *	0.001*	0.004*	
	**Percentage change**	6.38	9.05	7.45	

*OFD* Open flap Debridement, *L-PRF* Leukocyte platelet- rich fibrin, *L-PRF + CM* Leukocyte platelet- rich fibrin and collagen membrane

*PI* Plaque Index, *GI* Gingival index, *PD*  Probing depth, *CAL* Clinical attachment level

*CEJ-BD* Cemento Enamel Junction to Base of the Defect, *CEJ-AC* Cemento Enamel Junction to Alveolar Crest

*SD* standard deviation ( ±), *statistical significance 0.05%, ^a^ = Groups with statistical insignificance, ^b^ = Group with statistical significance, *NS* Non significant

Before surgical intervention, baseline periodontal indices (PI, GI, PPD, CAL) as well as radio-graphic parameters (CEJ-AC and CEJ-BD) demonstrated statistically insignificant differences between the three treatment groups (Table 1). Similarly, biochemical quantification of the two GF (PDGF and VEGF) on day 1 demonstrated statistically insignificant differences in mean concentrations between the three groups (Table 2).

**Table 2:** Mean, standard deviation and comparison of groups for biochemical outcomes

Growth Factor	Day	OFD (*n* = 10)	L-PRF (*n* = 10)	L-PRF + CM (*n* = 10)	Intergroup difference (*P-*value)
PDGF	1	1219.5 ± 187.5^a^	1130.6 ± 41.4^a^	1118.5 ± 69.8^a^	0.132 NS
	3	776.8 ± 106.6^b^	805.2 ± 80.0^b^	799.5 ± 85.7^b^	0.766 NS
	5	669.6^c^ ± 95.9	695.6^c^ ± 78.2	680.6^c^ ± 103.6	0.822 NS
	7	542.0 ± 122.5^d^	579.7 ± 76.2^d^	582.7 ± 104.7^d^	0.621 NS
	14	440.6 ± 122.1^e^	443.1 ± 56.1^e^	432.4 ± 86.8^e^	0.964 NS
	21	261.6 ± 88.8^f^	241.9 ± 58.8^f^	239.1 ± 71.7^f^	0.762 NS
	30	63.9 ± 25.3^ g^	77.3 ± 16.2^ g^	75.7 ± 10.6^ g^	0.223 NS
	***P-*value (Intragroup)**	< 0.001*	< 0.001*	< 0.001*	
	**Percentage change**	-94.7	-93.16	-93.2	
VEGF	1	1240.5 ± 14.8^a^	1242.5 ± 17.9^a^	1241.8 ± 17.4^a^	0.962 NS
	3	865.9 ± 6.1^b^	865.0 ± 5.6^b^	864.0 ± 6.6^b^	0.773 NS
	5	809.3^c^ ± 7.5	808.7^c^ ± 2.7	809.3^c^ ± 2.9	0.942 NS
	7	774.7 ± 7.5^d^	771.9 ± 9.5^d^	772.3 ± 10.6^d^	0.766 NS
	14	306.1 ± 2.2^e^	307.2 ± 2.0^e^	306.9 ± 2.1^e^	0.523 NS
	21	165.7 ± 2.2^f^	164.6 ± 2.1^f^	164.6 ± 2.0^f^	0.364 NS
	30	129.7 ± 14.8^ g^	129.4 ± 12.5^ g^	129.8 ± 12.4^ g^	0.998 NS
	***P-*value (Intragroup)**	< 0.001*	< 0.001*	< 0.001*	
	**Percentage change**	-89.54%	-89.58%	-89.54%	

Values for every sample = Mean concentration ± Standard deviation

*OFD* Open flap Debridement, *L-PRF*  Leukocyte platelet- rich fibrin, *L-PRF + CM*  Leukocyte platelet- rich fibrin and collagen membrane

*PDGF* Platelet derived growth factor, *VEGF* Vascular endothelial growth factor

*SD* Standard deviation ( ±), *statistical significance 0.05%, ^a−g^ = labels for days with statistical significance, *NS* Non significant

**Fig. 4:**
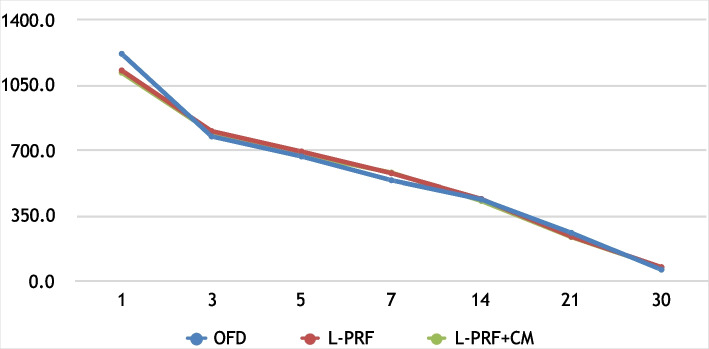
Line chart showing the mean PDGF decline over time for the three groups

**Fig. 5:**
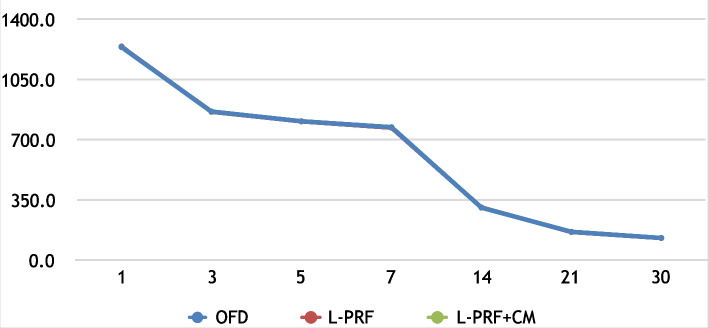
Line chart showing the mean VEGF for tested groups

Within each group, statistically significant reduction in the PI, GI, PD, and PD (*P* < 0.05) was observed throughout the study period. In a similar manner, the percentage of the alveolar crest (CEJ-AC) resorption was statistically significant in OFD (6.38%; *P* = 0.009), L-PRF (6.38%; *P* = 0.009), and L-PRF + CM (6.38%; *P* = 0.009). The percentage reduction in defect depth (CEJ-BD) was significant for OFD (7.25%; *P* = 0.001), L-PRF (12.6%; *P* = 0.001), and L-PRF + CM (32.6%; *P* = 0.001). As shown in Fig. 4, crevicular fluid samples showed statistically significant reduction in PDGF concentrations for OFD (94.7%; *P* = 0.001), L-PRF (93.16%; *P* = 0.001), and L-PRF + CM (93.2%; *P* = 0.001). Similarly, changes in VEGF (Fig. 5) concentrations was significant through time reaching a percentage reduc-tion of (89.54% (*P* = 0.001), 89.58% (*P* = 0.001), and 89.54% (*P* = 0.001) for OFD, L-PRF, and L-PRF + CM respectively. The lack of a significant reduction in PDGF and VEGF was only observed be-tween the 3rd and 5th day intervals in all three groups.

**Fig. 6:**
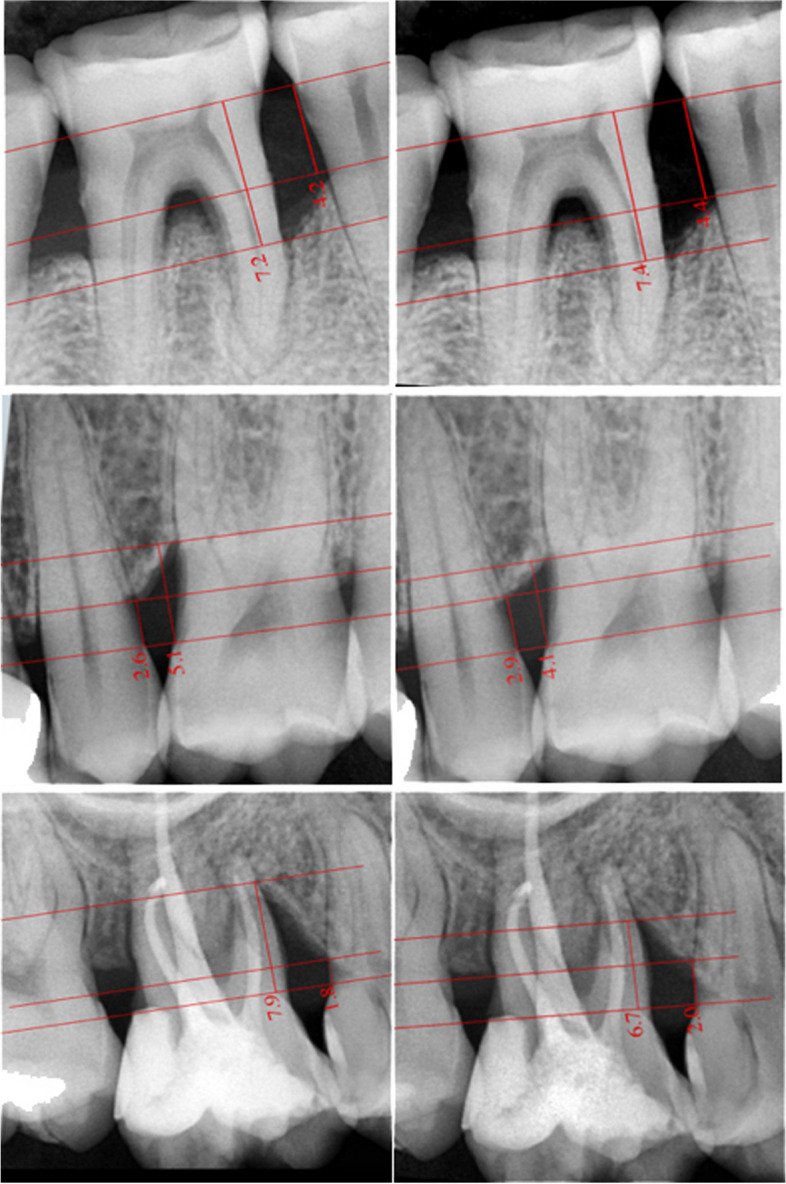
Radiographic views for the periodontal vertical defect at baseline (left) and at 6 months postoperative (right) for OFD (up left and right), L-PRF (middle left and right), and L-PRF + CM (below left and right)

Comparing between the three groups, changes in PI, GI, PD, and CAL were statistically insignificant after 6 months from surgical intervention (*P* > 0.05). While the amount of alveolar crest (CEJ-AC) resorption was not significant between the three groups (*P* = 0.24), reduction in the radiographic defect depth (CEJ-BD) was significantly greater (*P* = 0.001) in the third group (L-PRF + CM) (Fig. 6). Statistical comparison between the three groups for the concentrations of PDGF and VEGF revealed no significant differences in all measurement intervals (*P* > 0.05).

## Discussion

Periodontitis is a chronic inflammatory disease that is a leading cause of tooth loss in adults due to the progressive loss of supporting alveolar bone [12]. In regenerative periodontal therapy, the first strategy for successful restoration of periodontal defects applies GTR which involves protection of the infrabony periodontal lesion after debridement from penetration of the unfavorable gingival cells using barrier membranes. GTR have been extensively studied in the literature with successful clinical and experimental outcomes [13]. A second strategy in regeneration promotes the wound healing mechanism by triggering a triad of cells, signaling molecules, and a scaffold material [14].

The use of autologous platelet concentrates in the treatment of infrabony periodontal lesions have been reported in the literature with favorable results [15]. Among different platelet concentrates, L-PRF possesses a rigid fibrous scaffold acting as a reservoir for leukocytes and sustained release of signaling molecules. Unfortunately, little is known about the local GF release profile and the clinical equilibrium associated with its efficacy within the challenging periodontal defect [10]. Reviewing the literature, conflicting results existed between in vitro studies demonstrating the higher sustained concentration of GF in platelet concentrates [16, 17] and clinical trials reporting the lack of significant difference in release pattern and peak time of GF between unassisted and L-PRF assisted wounds [11, 18]. The latter claimed that even the increased initial GF concentrations with L-PRF did not translate into clinical benefits [18]. Interestingly CM are not considered mere passive barriers in periodontal wounds but active contributors in progenitor cell adhesion, and chemotaxis as well as GF adsorption. Among various studies about release kinetics of GF on barrier membranes, PDGF was studied most [19].

The contradiction in GF release between in- vitro and clinical studies as well as the reported adsorptive/ release potential of loaded CM arose the hypothesis of the possible benefit of using CM in retaining higher levels of GF from L-PRF harvests within intrabony defects. In the present study, coverage of L-PRF filled intrabony periodontal lesions with CM is investigated clinically and radiographically. In addition, the hypothesis for the local benefit of using CM in preserving PDGF-BB and VEGF concentrations is addressed.

The patients enrolled in the current study had stage III periodontitis with interproximal 2 or 3 osseous wall contained defects that are ≥ 3mm in depth. Predictability of regeneration depends not only on the number of remaining osseous walls but also the defect depth; deeper defects with higher number of osseous walls demonstrated higher radiographic bone gain and improved CAL [20]. Of all GF studied within L-PRF, PDGF and VEGF were the GF of choice for biochemical assessment. PDGF gained much attention in the literature due to its significant role in initiating the wound healing process, strengthening the wounds by collagen content elevation, and the stimulation of fibroblastic proliferation and migration [21]. VEGF gained much interest due to its role in controlling tissue perfusion and angiogenesis via enhancing vascular permeability and vascular endothelial cell chemotaxis [6].

In the current study, a statistically significant drop in GI and PI within every group was recorded despite the comparison between the tests and control was statistically insignificant. This suggests that oral hygiene measures and local factors debridement had a higher influence over the gingival health than the treatment protocol. Such findings run parallel with Sharma and Pradeep who reported a statistically significant intragroup reduction in PI and bleeding index without statistically significant difference between OFD and PRF in the treatment of furcation defects over a period of 9 months [22].

The mean PD reduction following treatment was ⁠3.8, 3.8 and 3.9 mm for OFD, L-PRF, and L-PRF + CM respectively. Despite L-PRF + CM group highest reduction in PD, there was no statistically significant intergroup difference. Similarly, the CAL mean reductions were 3.1mm, 3 mm and 2.6 mm for the three groups respectively in order with statistically insignificant difference between them despite the statistically significant reduction within all groups between observation intervals. This corresponds with one study which reported the lack of a statistically significant difference between CM and L-PRF in PD and CAL reduction [23]. The questionable benefit of L-PRF use in improving PD and CAL of infrabony defects coincide with a study reporting no statistically significant reduction in PD and CAL when L-PRF was added to Xenografts in comparison to the sole use of Xenografts or PRGF [11]. The conflicting results between the present study with the others [24] may be attributed to the heterogeneity in design and follow up range. All the screened studies lacked a standardized protocol for L-PRF preparation which complicates an accurate interpretation and comparison between results. Based on this, Castro et al. recommended directing the future research and manufacturers towards standardization of the preparation protocol and producing machinery [25].

The radiographic outcomes for the L-PRF + CM group demonstrated a significantly greater reduction in DBF mean distance (2.12 mm) and percentage change (32.6%) in comparison to the OFD (mean reduction: 0.43 mm, % change: 7.25%) and L-PRF group (mean reduction: 0.8 mm, % change: 12.6%). The grounds of this reduction could be the expected synergistic effect of the GTR and the protected L-PRF membranes where the L-PRF expressed its classical potentials as GTR membranes. One clinical trial reported a greater percentage reduction (58.19 ± 13.24%) in defect depth fill when CM was used with PRF in comparison to CM [26]. Regarding the changes in the AC level assessment, all groups showed statistically significant intragroup mean reduction throughout the 6 months period unlike the interior comparisons which showed insignificant statistical significance. In the present study, one non tested interchangeable factor that may influence the amount of AC resorption is the surgical approach and flap design. Several theories are found to justify the alveolar crest reduction following flap reflection; First is the raised osteoclastic activity following mucoperiosteal flap elevation [27], a second explanation namely strain relaxation hypothesis claimed a relaxation of tension in gingival collagen fibers after severing them which stimulates alveolar bone resorption [28]. The same theory is supported by evidence of observing the rounding up of fibroblasts possessed by the periodontal ligament fibers following the mucoperiosteal flap surgeries, [28] whereas they tend to take a less elongated morphology in periodontitis models [29]. The non-significant difference in the amount of post-operative crestal resorption suggests failure of L-PRF in neutralizing the negative effects of the factors contributing to resorption.

For the growth factors’ concentrations, having the peak level at the first day following surgical intervention with subsequent regression was consistent with the existing evidence in the literature [10]. The sustainable reduction in PDGF levels throughout the 30 day period is also consistent with the findings of one study, which evaluated the PDGF levels in infrabony lesions treated with L-PRF membranes in comparison to CM having recombinant PDGF-BB [30]. Another study demonstrated a continuous decline of PDGF and VEGF despite the initial progression in the first three days [17]. Measurements obtained and sample preparation through incubation in culture in the latter study may justify the initial difference in release profile from the current study. Moreover, extraction wounds failed to demonstrate a difference in the release pattern of GF between L-PRF wounds versus the wounds with unassisted healing [10]. Several possible explanations clarify the lack of difference in GF between treatment protocols; (a) the diluting effect of the gingival crevicular fluid for the claimed high levels of GF released from L-PRF membrane in the local environment; (b) the open wound nature of periodontal defects; (c) the persistently contaminated nature of periodontal defects loaded with catalytic bacterial enzymatic activity; (d) the presence of a barrier membrane itself may have segregated the deeper GF concentrations from those in the crevicular fluid above it; (e) the use of conventional flap design rather than papilla preservation flaps might have compromised the degree of wound closure and, consequently, the amount of bone fill and clinical attachment gain. Bearing in mind that the biochemical sample is obtained in a standardized fashion using filter paper strips at a constant subgingival depth, it is possible that a different concentration of GF existed beneath the CM that was inaccessible to paper strips during sampling, and that such masked release might have built the grounds for the statistically significant gain in hard tissue fill for the L-PRF + CM group.

One limitation that might have affected the clinical outcomes was the gender variation among the groups. Reviewing the literature, there is a lack in evidence for the influence of gender on the amount of radiographic bone fill following regenerative therapy [31, 32].

On the molecular level, gender differences raise a question for the possible structural and physiologic differences in L-PRF harvests between the groups. The literature demonstrates that the fewer erythrocytic count in female and older patients were associated with larger size PRF membranes. The same study hypothesized that larger membranes might be associated with higher density of cellular elements and signaling molecules [33].

The results of the present study contrasts the possible influence of gender type on the release profile of PDGF and VEGF. This conclusion should be carefully interpreted since we only evaluated two GF. All patients were prescribed Ibuprofen following surgical intervention for the management of pain and inflammation. Therefore, it was considered the drug pharmacodynamics and their possible influence on the secretory profile of GF. Under inflammatory conditions, one study demonstrated statistically significant reduction in endothelial cell viability and proliferation as well as an increase in VEGF production following the administration of Ibuprofen [34].

## Conclusions

Within the limitations of this study, the present data seem to support the effectiveness of using GTR membranes in association with L-PRF for improving the intrabony defect fill. On a bio-molecular level, periodontal defect ecology failed to maintain any L-PRF claimed extra physiologic concentration of growth factors (PDGF and VEGF) even following collagen barrier membrane protection. Future studies with larger sample size as well as testing other subtypes of PRF are recommended.

### Supplementary Information

**Additional file 1: Supplementary Table 1. **Summary for demographic data.

## Data Availability

The data that support the findings of this study are available from the corresponding author upon reasonable request.

## Abbreviations

L-PRF: Leukocyte platelet-rich fibrin
GF: Growth factors
CM: Collagen membrane
OFD: Open flap debridement
PI: Plaque index
GI: Gingival index
PD: Probing depth
CAL: Clinical attachment level
DBF: Radiographic defect base fill
PDGF-BB: Platelet Derived Growth Factor-BB
VEGF: Vascular Endothelial Growth Factors
GTR: Guided tissue regeneration
AC: Interproximal alveolar crest
BD: Base of the defect
CEJ: Cemento-enamel junction
PRGF: Plasma rich in growth factors

## Footnotes

1
www.randomizer.org

2 Hu-Friedy Mfg. Co., LLC, USA.

3 Artinibsa 2% Inibsa Dental S.L.U, Ctra. Sabadell a Granollers, KM 14,5 (C-155), 08185 Lliçà de Vall (Barcelona), Spain.

4 Silicon-coated interior, Intraspin®; IntraLockTM, Boca Raton, FL, USA.

5 Intraspin®; IntraLockTM, Boca Raton, FL, USA.

6 Pearson Dental, Sylmar, Ca

7 Tutopatch RTI Surgical Inc, FL, USA.

8 Brufen 400 mg; Kahira Pharm. & Chem. Ind. Co., Under license from: Abbott Laboratories.

9 Floxamo 500 mg; Amoun Pharmaceutical Company S.A.E. Cairo – Egypt.

10 Hexitol; Kahera Pharmaceutical, Cairo, Egypt.

11 New IDA, Eagle, Brazil.

12 Preva Dental X-ray System, Progeny, Midmark, Illinois, USA.

13 DabiAtlante, Sao Paulo, Brazil.

14 Oraflow Inc., New York, United States.

15 RayBiotech, Norcross, GA.

16 Microsoft Excel 2016®, DC, USA.

17 IBM SPSS Statistics for Windows, version 20.0. Armonk, NY: IBM Corp, USA.

18 Graphpad Software, Boston, USA.
