# Supplementary material for: Adjunctive effect of collagen membrane coverage to L-PRF in the treatment of periodontal intrabony defects: a randomized controlled clinical trial with biochemical assessment
Source: BMC Oral Health. 2023 Sep 4;23:631. doi: 10.1186/s12903-023-03332-0 (PMC10476412; doi:10.1186/s12903-023-03332-0)
Supplement: Supplementary file 1 — Additional file 1: Supplementary Table 1. Summary for demographic data. [file 12903_2023_3332_MOESM1_ESM.docx]

**Supplemental appendix:**

**S Table (1): Summary for demographic data:**

|  | OFD (n = 10) | | L-PRF (n = 10) | | L-PRF +CM (n = 10) | |
| --- | --- | --- | --- | --- | --- | --- |
|  | Mean | SD | Mean | SD | Mean | SD |
| Age | 38.3 | 4.1 | 42.2 | 2.9 | 41.6 | 4.3 |
| Gender | 80% males | | 30%. males | | 10% males | |
| Defect morphology |  |  |  |  |  |  |
| *Two wall defects* | 6 | | 7 | | 3 | |
| *Three wall defects* | 4 | | 3 | | 7 | |
